# A double projection algorithm for quasimonotone variational inequalities in Banach spaces

**DOI:** 10.1186/s13660-018-1852-2

**Published:** 2018-09-24

**Authors:** Lian Zheng

**Affiliations:** Department of Mathematics and Statistics, Yangtze Normal University, Chongqing, China

**Keywords:** 90C25, 90C30, Variational inequalities, Banach spaces, Double projection algorithm, Quasimonotone, Convergence

## Abstract

We propose a double projection algorithm for solving variational inequality problems in Banach spaces. We establish the strong convergence of the whole sequence generated by the proposed method under the quasimonotone and uniform continuity on bounded sets, which are weaker conditions than those used in existing projection-type methods for solving variational inequality problems in Banach spaces.

## Introduction

Let $\mathbb{B}$ be a reflexive Banach spaces with norm $\| \cdot\|$, and let $\mathbb{B^{*}}$ be its topological dual with norm $\|\cdot\|_{*}$. By $\langle x^{*},x\rangle$ we denote the duality coupling in $\mathbb{B^{*}}\times\mathbb{B}$ defined by $\langle f,x\rangle=f(x)$ for all $x\in\mathbb{B}$ and $f\in\mathbb {B^{*}}$. By $x_{n}\rightarrow x$ and $x_{n}\rightharpoonup x$ we denote the strong and weak convergence of a sequence $\{x_{n}\}$ to *x*, respectively. We consider the following variational inequality problem, denoted by $\operatorname{VI}(T,C)$: find a vector $x^{*}\in C$ such that

1 $$ \bigl\langle T\bigl(x^{*}\bigr),y-x^{*}\bigr\rangle \ge0 \quad \text{for all }y\in C, $$

where *C* is a nonempty closed convex subset of $\mathbb{B}$, and $T: \mathbb{B}\rightarrow\mathbb{B^{*}}$ is an operator.

Let *S* be the solution set of $\operatorname{VI}(T,C)$, and let $S_{D}$ be the solution set of the dual variational inequality, that is,

$$S_{D}:=\bigl\{ x\in C|\bigl\langle T(y),y-x\bigr\rangle \geq0 \text{ for all } y\in C \bigr\} . $$

If *T* is continuous and *C* is convex, then we have

$$S_{D}\subset S. $$

Indeed, for any $\tilde{x} \in S_{D}$, we have $\tilde{x} \in C$. For any given $y \in C$ and $t\in[0,1]$, applying the convexity of *C*, we obtain

$$(1-t)\tilde{x} +ty \in C. $$

Therefore the definition of $S_{D}$ implies that

$$\bigl\langle T\bigl((1-t)\tilde{x}+ty\bigr),(1-t)\tilde{x}+ty-\tilde{x}\bigr\rangle \geq0 $$

or, equivalently,

$$\bigl\langle T\bigl((1-t)\tilde{x}+ty\bigr),y-\tilde{x}\bigr\rangle \geq0. $$

Letting $t\rightarrow0$, by the continuity of *T* we obtain

$$\bigl\langle T(\tilde{x}),y-\tilde{x} \bigr\rangle \geq0, $$

that is, $\tilde{x}\in S$, and thus, $S_{D}\subset S$.

The variational inequality problem was first introduced by Hartman and Stampacchia [1] in 1966. The projection-type algorithms for solving the variational inequality problem have been extensively studied in a finite-dimensional space, such as proximal point methods [2], extragradient projection methods [3–6], double projection methods [7–10], and self-adaptive projection methods [11, 12]. To prove the convergence of a generated sequence, all the methods mentioned have the common assumption $S\subset S_{D}$, that is,

2 $$ \bigl\langle T(y), y-x^{*}\bigr\rangle \geq0\quad \text{for all }x^{*}\in S \mbox{ and } y\in C, $$

which is a direct consequence of the pseudomonotonicity of *T* on *C* in the sense of Karamardian [13]; *T* is said to be pseudomonotone on *C* if for all $x,y \in C$,

$$\bigl\langle T(x),y-x\bigr\rangle \geq0\quad \Rightarrow\quad \bigl\langle T(y),y-x\bigr\rangle \geq0; $$

*T* is said to be quasimonotone on *C* if for all $x,y \in C$,

$$\bigl\langle T(x),y-x\bigr\rangle > 0\quad \Rightarrow\quad \bigl\langle T(y),y-x \bigr\rangle \geq0. $$

Note that pseudomonotone implies quasimonotone, but the converse is not true.

Recently, in the literature [14, 15],an interior proximal algorithm for solving quasimonotone variational inequalities is proposed, and the global convergence is obtained under more assumptions than $S_{D}\neq \emptyset$ and quasimonotonicity. Clearly,

3 $$ S_{D}\neq\emptyset\quad \Longleftrightarrow\quad \exists x^{*}\in S\quad \text{such that}\quad \bigl\langle T(y), y-x^{*} \bigr\rangle \geq0 \quad \text{for all } y\in C, $$

and $S_{D}\neq\emptyset$ is weaker than assumption (2). Thus $S\neq \emptyset$ and pseudomonotonicity contain quasimonotonicity and $S_{D}\neq\emptyset$, whereas the converse implications are not true. For sufficient conditions for $S_{D}\neq\emptyset$, see Lemma 2.6.

On the other hand, recently, in [14–16] an extragradient-type method proposed in [5] is extended from Euclidean spaces to Banach spaces. Under the assumptions of the pseudomontonicity, uniform (or strong) continuity, and $S\neq\emptyset$, the global strong convergence is obtained. In [17] a double projection method in Banach space is studied, and the global weak convergence is obtained under more assumptions than the pseudomontonicity and uniform continuity.

Inspired by the works mentioned, in this paper, by Bregman projection we extend a double projection algorithm proposed by Solodov and Svaiter [7] for solving variational inequalities from Euclidean spaces to Banach spaces. Under the assumptions of $S_{D}\neq\emptyset$, uniform continuity, and quasimonotonicity, we prove that the whole sequence generated by the proposed method is strongly convergent to the solution of the variational inequalities, and our proof techniques are different from those presented in [14–17].

## Preliminaries

In this section, we recall some useful definitions and results. First, we state some properties of the Bregman distance taken from [18].

### Definition 2.1

Let $g:\mathbb{B}\rightarrow\mathbb{R}$ be a Gâteaux-differentiable function.

(i) The Bregman distance with respect to *g* is the function $D_{g}:\mathbb{B}\times\mathbb{B}\rightarrow\mathbb{R}$ defined as
  $$D_{g}(x,y)=g(x)-g(y)-\bigl\langle g'(y),x-y\bigr\rangle , \quad x,y\in\mathbb{B}, $$
  where
  $$\bigl\langle g'(y),x-y\bigr\rangle =\lim_{t\rightarrow0} \frac{g(y+t(x-y))-g(y)}{t}. $$
(ii) The modulus of total convexity of *g* at the point $x\in \mathbb{B}$ is the function $\nu_{g}:\mathbb{B}\times[0,+\infty )\rightarrow[0,+\infty)$ defined as
  $$\nu_{g}(x,t)=\inf\bigl\{ D_{g}(x,y):y\in\mathbb{B},\|y-x\|=t \bigr\} . $$
(iii) A function *g* is said to be totally convex if $\nu _{g}(x,t)>0$ for all $t>0$ and $x\in\mathbb{B}$.
(iv) *g* is said to be a strongly convex function if there exists $\alpha>0$ such that
  $$g(x)-g(y)-\bigl\langle g'(y),x-y\bigr\rangle \geq\alpha\|x-y \|^{2},\quad \forall x,y\in\mathbb{B}. $$

### Remark 2.1

1. It should be noted that $D_{g}$ is not a distance in the usual sense of the term. In general, $D_{g}$ is not symmetric and does not satisfy the triangle inequality. Clearly, $D_{g}(x,x)=0$, but $D_{g}(x,y)=0$ may not imply $x = y$, for instance, when *g* is a linear functional on $\mathbb{B}$. If *g* is strictly convex or strongly convex on $\mathbb{B}$, then we have that $D_{g}(x,y)> 0$ for $x,y\in\mathbb{B}$, $x\neq y$.
2. Clearly, if *g* is a strongly convex function, then *g* is a totally convex function.
3. If $g(x)=\frac{1}{2}\|x\|^{2}$ and $\mathbb{B}$ is a Hilbert space, then $D_{g}(x,y)=\frac{1}{2}\|x-y\|^{2}$.

We present some conditions on an auxiliary function, called *g*, which are important for the feasibility and the convergence analysis of our algorithm.

1. The level sets of $D_{g}(x,\cdot)$ are bounded for all $x\in \mathbb{B}$.
2. *g* is strongly convex on $\mathbb{B}$.
3. $g'$ is uniformly continuous on bounded subsets of $\mathbb{B}$.
4. $g'$ is onto, that is, for all $y\in\mathbb{B^{*}}$, there exists $x\in\mathbb{B}$ such that $g'(x)=y$.
5. $(g')^{-1}$ is uniformly continuous on bounded subsets of $\mathbb{B^{*}}$.

On the feasibility of the assumptions (H1)–(H5), see [17, 19, 20] and the references therein. If $\mathbb{B}=\mathbb{R}$, then $g(x)=x^{2}$ satisfies assumptions (H1)–(H5).

We recall the definition of the Bregman projection and some useful results.

### Lemma 2.1

*Assume that* $\mathbb{B}$ *is a Banach space*, *C* *is a nonempty*, *closed*, *and convex subset of* $\mathbb{B}$, $g: \mathbb{B}\rightarrow \mathbb{R}$ *is a totally convex function on* $\mathbb{B}$ *satisfying* (H1). *Then there exists unique* $\hat{x}\in C$ *such that* $\hat{x}=\min_{x\in C}D_{g}(x,\bar{x})$; *x̂* *is called the Bregman projection of* *x̄* *onto* *C* *and is denoted by* $\Pi^{g}_{C}(\bar{x})$, *and* $\hat{x}=\Pi^{g}_{C}(\bar{x})$ *if and only if* $g'(\bar{x})-g'(\hat {x})\in N_{C}(\hat{x})$ *or*, *equivalently*, *if* $\hat{x}\in C$ *and*

$$\bigl\langle g'(\bar{x})-g'(\hat{x}),y-\hat{x}\bigr\rangle \leq0,\quad \forall y\in C. $$

### Proof

See p. 70 of [18]. □

### Lemma 2.2

*Assume that* (H2) *is satisfied*. *Let* $\{x^{k}\}$ *and* $\{y^{k}\}$ *be two sequences of* $\mathbb{B}$ *such that at least one of them is bounded*. *If*

$$\lim_{k\rightarrow\infty}D_{g}\bigl(y^{k},x^{k} \bigr)=0, $$

*then*

$$\lim_{k\rightarrow\infty}\bigl\| x^{k}-y^{k}\bigr\| =0. $$

### Proof

See Proposition 5 of [19]. □

### Lemma 2.3

*Let* $C\subseteq\mathbb{B}$ *be a nonempty*, *closed*, *and convex subset*, *let* *U* *be a bounded subset of* $\mathbb{B}$, *and let* $g: \mathbb {B}\rightarrow R$ *be a totally convex and Fréchet*-*differentiable function*. *If* (H1) *and* (H3) *hold*, *then* $\Pi ^{g}_{C}:\mathbb{B}\rightarrow C$ *maps* *U* *into a bounded subset of* *C*.

### Proof

See Proposition 2.10 of [20]. □

### Lemma 2.4

*Let* $\mathbb{B}_{1}$ *and* $\mathbb{B}_{2}$ *be Banach spaces*. *Let* *U* *be a bounded subset of* $\mathbb{B}_{1}$. *If* $T:\mathbb {B}_{1}\rightarrow\mathbb{B}_{2}$ *is uniformly continuous on bounded subsets of* $\mathbb{B}_{1}$, *then* *T* *is bounded on* *U*.

### Lemma 2.5

*Let* *T* *be a continuous and quasimonotone operator*, *and let* $y\in C$. *If for some* $x_{0}\in C$, *we have* $\langle T(y),x_{0}-y\rangle\geq0$, *then at least one of the following must hold*:

$$\bigl\langle T(x_{0}), x_{0}-y\bigr\rangle \geq0 \quad \textit{or}\quad \bigl\langle T(y), x-y\bigr\rangle \leq0,\quad \forall x\in C. $$

### Proof

See Lemma 3.1 of [21]. □

### Lemma 2.6

*If either*

(i) *T* *is pseudomonotone on* *C*, *and* $S\neq\emptyset$;
(ii) *T* *is the gradient of* *G*, *where* *G* *is a differentiable quasiconvex function on an open set* $K\supset C$ *and attains its global minimum on* *C*;
(iii) *T* *is quasimonotone on* *C*, $F\neq0$, *and* *C* *is bounded*;
(iv) *T* *is quasimonotone on* *C*, $F\neq0$, *and there exists a positive number* *r* *such that*, *for every* $x\in C$ *with* $\|x\|\geq r$, *there exists* $y\in C$ *such that* $\|y\|\geq r$ *and* $\langle T(x),y-x\rangle\leq0$;
(v) *T* *is quasimonotone on* *C*, *intC* *is nonempty*, *and there exists* $x^{*}\in S$ *such that* $T(x^{*})\neq\emptyset$, *then* $S_{D}\neq\emptyset$.

### Proof

See Proposition 2.1 of [22]. □

### Definition 2.2

$T:\mathbb{B}\rightarrow\mathbb{B^{*}}$ is said to be

(i) strongly continuous at a point *x* if for $\{x_{n}\}\subset D(T)$, from $x_{n}\rightharpoonup x$ it follows that $T(x_{n})\rightarrow T(x)$;
(ii) continuous at a point *x* if for $\{x_{n}\}\subset D(T)$, from $x_{n}\rightarrow x$ it follows that $T(x_{n})\rightarrow T(x)$;
(iii) uniformly continuous on a subset *K* of $D(T)$ if for all $\varepsilon>0$, there exists $\delta>0$ such that, for $x,y\in K$, from $\|x-y\|<\delta$ it follows that $\|T(x)-T(y)\|_{*}<\varepsilon$;
(iv) (strongly) continuous on $D(T)$ if *T* is (strongly) continuous at each point of $D(T)$.

### Remark 2.2

We can see that the strong continuity and the uniformly continuity are two different concepts, and they both contain the continuity, whereas the converse implications are not true. Under the assumptions of the strong continuity and pseudomonotonicity, in [16] the convergence of the sequence produced is proved.

## Algorithm and feasibility analysis

### Algorithm 3.1

Choose $x^{0}\in C$ and two parameters: $\gamma, \sigma\in (0,1)$. Take $k=0$.

Step 1. Compute
  $$z^{k}=\bigl(g'\bigr)^{-1}\bigl[g' \bigl(x^{k}\bigr)-T\bigl(x^{k}\bigr)\bigr]. $$
  If $x^{k}=\Pi^{g}_{C}(z^{k})$, then stop; else go to Step 2.
Step 2. Compute
  4 $$ m_{k}=\text{min}\bigl\{ m\in\mathbb{N}:\bigl\langle T \bigl(x^{k}\bigr)-T\bigl(y^{m}\bigr), x^{k}- \Pi^{g}_{C}\bigl(z^{k}\bigr)\bigr\rangle \leq\sigma D_{g}\bigl(\Pi ^{g}_{C}\bigl(z^{k} \bigr),x^{k}\bigr)\bigr\} , $$
  where
  $$y^{m}=\gamma^{m}\Pi^{g}_{C} \bigl(z^{k}\bigr)+\bigl(1-\gamma^{m}\bigr)x^{k}. $$
  Let
  $$\alpha_{k}=\gamma^{m_{k}}, y^{k}=\alpha_{k} \Pi ^{g}_{C}\bigl(z^{k}\bigr)+(1- \alpha_{k})x^{k}. $$
Step 3. Compute
  $$x^{k+1}=\Pi^{g}_{C\cap H_{k}}\bigl(x^{k}\bigr), $$
  where
  5 $$ \begin{aligned} &\mathrm{H}_{k}=\bigl\{ \nu:h_{k}(\nu)\leq0\bigr\} , \\ &h_{k}(\nu)=\bigl\langle T\bigl(y^{k}\bigr), \nu-y^{k}\bigr\rangle . \end{aligned} $$
Step 4. Let $k: =k+1$ and return to Step 1.

The feasibilities of Step 1 and Step 2 of the Algorithm 3.1 are explained in the following:

### Lemma 3.1

*If* $g:\mathrm{B}\rightarrow R$ *satisfies* (H1)*–*(H4), *then Step* 1 *and Step* 2 *of Algorithm* 3.1 *are well defined*.

### Proof

For given $x^{k}\in C$, the feasibility of $z^{k}$ follows from (H4). If $x^{k}=\Pi^{g}_{C}(z^{k})$, then it follows from Lemma 2.1 that $x^{k}$ is a solution of $\operatorname{VIP}(T,C)$. If $\Pi^{g}_{C}(z^{k})\neq x^{k}$, then Step 2 of the algorithm is well defined; otherwise, for all nonnegative integers *m*, we have

6 $$ \bigl\langle T\bigl(x^{k}\bigr)-T\bigl(y^{m} \bigr),x^{k}-\Pi^{g}_{C}\bigl(z^{k}\bigr) \bigr\rangle >\sigma D_{g}\bigl(\Pi^{g}_{C} \bigl(z^{k}\bigr),x^{k}\bigr). $$

Since $\gamma\in(0,1)$, we have

$$\lim_{m\rightarrow\infty}y^{m}=\lim_{m\rightarrow\infty}\bigl[ \gamma ^{m}\Pi^{g}_{C}\bigl(z^{k}\bigr)+ \bigl(1-\gamma^{m}\bigr)x^{k}\bigr]=x^{k}. $$

Now letting $m\rightarrow\infty$ in (6), by $\sigma>0$ and the continuity of *T* we obtain that

$$D_{g}\bigl(\Pi^{g}_{C}\bigl(z^{k} \bigr),x^{k}\bigr)\leq0. $$

Note that $\Pi^{g}_{C}(z^{k})\neq x^{k}$ and *g* is strongly convex, which implies that $D_{g}(\Pi^{g}_{C}(z^{k}),x^{k})>0$, a contradiction. So $m^{k}$, $\alpha^{k}$, and $y^{k}$ are well defined. □

The following lemma shows that Step 3 of Algorithm 3.1 is also feasible.

### Lemma 3.2

*For all* $x\in C$, *we have*

7 $$ \bigl\langle T(x),x-\Pi^{g}_{C}\bigl[ \bigl(g'\bigr)^{-1}\bigl(g'(x)-T(x)\bigr)\bigr] \bigr\rangle \geq D_{g}\bigl(\Pi^{g}_{C}\bigl[ \bigl(g'\bigr)^{-1}\bigl(g'(x)- T(x)\bigr) \bigr],x\bigr). $$

### Proof

See Lemma 2.5 of [16]. □

### Remark 3.1

We apply Lemma 3.2 and (4) to obtain

$$\bigl\langle T\bigl(y^{k}\bigr), x^{k}-\Pi^{g}_{C} \bigl(z^{k}\bigr)\bigr\rangle \geq(1-\sigma)D_{g}\bigl(\Pi ^{g}_{C}\bigl(z^{k}\bigr),x^{k}\bigr). $$

Then taking $\mathbb{B}=\mathbb{R}^{n}$, $\sigma:=1-\sigma$, and $g(x)=\frac{1}{2}\|x\|^{2}$ in Algorithm 3.1, our Algorithm 3.1 degrades into Algorithm 2.2 of [7].

### Lemma 3.3

*Assume that* $S_{D}$ *is nonempty and* $\{x^{k}\}$ *is generated by Algorithm* 3.1. *Then* $S_{D}\subset C\cap H_{k}$, *and*

8 $$ h_{k}\bigl(x^{k}\bigr)\geq \alpha_{k}(1-\sigma)D_{g}\bigl(\Pi^{g}_{C} \bigl(z^{k}\bigr),x^{k}\bigr). $$

### Proof

Applying Remark 3.1, $\alpha_{k}>0$, and $\sigma\in(0,1)$, we have

$$\begin{aligned} h_{k}\bigl(x^{k}\bigr) =&\bigl\langle T\bigl(y^{k} \bigr),x^{k}-y^{k}\bigr\rangle \\ =& \alpha_{k}\bigl\langle T\bigl(y^{k}\bigr),x^{k}- \Pi^{g}_{C}\bigl(z^{k}\bigr)\bigr\rangle \\ \geq& \alpha_{k}(1-\sigma) D_{g}\bigl( \Pi^{g}_{C}\bigl(z^{k}\bigr),x^{k} \bigr)>0. \end{aligned}$$

For all $x^{*}\in S_{D}$, we have $\langle T(y^{k}),x^{*}-y^{k}\rangle\leq0$, from which it follows that $x^{*}\in C\cap H_{k}$, so $S_{D}\subset C\cap H_{k}\neq\emptyset$. □

### Remark 3.2

Clearly, $C\cap H_{k}$ is closed and convex. It follows from Lemma 2.1 that the generation of the iteration point $x^{k+1}$ in Step 3 is feasible. So Step 3 is well defined. By Lemma 3.3 we know that the hyperplane $H_{k}$ strictly separates the current iterate from the solutions of $\operatorname{VI}(T,C)$.

### Lemma 3.4

*If* $x^{k}\neq\Pi^{g}_{C}(z^{k})$, *then* $T(x^{k})\neq0$.

### Proof

Since $x^{k}\neq\Pi^{g}_{C}(z^{k})$, by Lemma 2.1 there exists $y_{0}\in C$ such that

$$ \bigl\langle g'\bigl(z^{k}\bigr)-g' \bigl(x^{k}\bigr),y_{0}-x^{k}\bigr\rangle >0. $$

By the definition of $z^{k}$ we obtain

$$ \bigl\langle g'\bigl(x^{k}\bigr)-T\bigl(x^{k} \bigr)-g'\bigl(x^{k}\bigr),y_{0}-x^{k} \bigr\rangle =\bigl\langle -T\bigl(x^{k}\bigr),y_{0}-x^{k} \bigr\rangle >0, $$

which implies $T(x^{k})\neq0$. □

### Lemma 3.5

*Let* *C* *be a closed convex subset of* $\mathbb{B}$, *and let* *g* *be a continuously differentiable function satisfying* (H1) *and* (H2). *Define* $h:\mathbb{B}\times\mathbb{B}\rightarrow\mathbb{R}$ *by* $h(x,v)=\langle T(v),x-v\rangle$ *for any given* $v\in\mathbb{B}$ *and take* $K(v)=\{x\in C:h(x,v)\leq0\} $. *If* $K(v)\neq\emptyset$ *and* $h(\cdot,\cdot)$ *is Lipschitz continuous with respect to the first variable on* *C* *with modulus* $L>0$, *then*

9 $$ D_{g}(x,y)\geq\frac{\alpha}{L^{2}}h^{2}(x,v),\quad \forall x\in C\setminus K(v), y\in K(v), v\in\mathbb{ B}. $$

### Proof

First, we prove that, for all $v\in\mathbb {B}$, $K(v)$ is a convex set. In fact, for all $x_{1},x_{2}\in K(v)$ and $\theta\in(0,1)$, we have

$$\begin{aligned}& h(x_{1},v)\leq0,\qquad h(x_{2},v)\leq0, \\& \begin{aligned} h\bigl(\theta x_{1} +(1-\theta)x_{2},v \bigr)&=\bigl\langle T(v),\theta x_{1} +(1-\theta)x_{2}-v \bigr\rangle \\ &=\bigl\langle T(v),\theta(x_{1}-v)\bigr\rangle +\bigl\langle T(v),(1- \theta ) (x_{2}-v)\bigr\rangle \\ &=\theta\bigl\langle T(v), x_{1}-v\bigr\rangle +(1-\theta)\bigl\langle T(v),x_{2}-v\bigr\rangle \\ &=\theta h(x_{1},v)+(1-\theta)h(x_{2},v)\leq0. \end{aligned} \end{aligned}$$

So $\theta x_{1} +(1-\theta)x_{2}\in K(v)$, and $K(v)$ is convex. Since *h* is continuous, we conclude that $K(v)$ is also a closed set. For all $x\in C\setminus K(v)$, it follows from (H1), (H2), and Lemma 2.1 that there exists unique $y(x)\in K(v)$ such that

$$D_{g}\bigl(x,y(x)\bigr)=\min_{y\in K(v)}D_{g}(x,y). $$

By the definition of $K(v)$ and the Lipschitz continuity of $h(\cdot ,\cdot)$ with respect to the first variable on *C*, we obtain

10 $$ h(x,v)\leq h(x,v)-h\bigl(y(x),v\bigr)= \bigl\vert h(x,v)-h \bigl(y(x),v\bigr) \bigr\vert \leq L \bigl\Vert x-y(x) \bigr\Vert . $$

Since *g* is strongly convex, there exists $\alpha>0$ such that, for all $x,y\in\mathbb{B} $,

$$g(x)-g(y)-\bigl\langle g'(y),x-y\bigr\rangle \geq\alpha\|x-y \|^{2}, $$

that is,

$$\begin{aligned}& D_{g}(x,y)\geq\alpha\|x-y\|^{2}, \\& \|x-y\|\leq\sqrt{\frac{D_{g}(x,y)}{\alpha}}, \end{aligned}$$

which by (10) implies that

$$h(x,v)\leq L \bigl\Vert x-y(x) \bigr\Vert \leq L\sqrt{\frac{D_{g}(x,y(x))}{\alpha}}\leq L\sqrt{\frac{D_{g}(x,y)}{\alpha}}, \quad \forall y\in K(v). $$

Thus

$$D_{g}(x,y)\geq\frac{\alpha}{L^{2}}h^{2}(x,v), \quad \forall x \in C\setminus K(v), y\in K(v), v\in\mathbb{B}. $$

 □

## The convergence of algorithm

### Theorem 4.1

*Assume that* $S_{D}$ *is a nonempty set*, $T:\mathbb{B}\rightarrow \mathbb{B^{*}}$ *is uniformly continuous on bounded subsets of* $\mathbb {B}$, *and* $g:\mathrm{B}\rightarrow R$ *satisfies* (H1)*–*(H5). *If* *C* *is a closed and convex subset of* *B* *and* $\{x^{k}\}$ *is an infinite sequence generated by Algorithm* 3.1, *then*

(i) 11 $$ D_{g}\bigl(x^{k+1},x^{k}\bigr)\leq D_{g}\bigl(x^{*},x^{k}\bigr)-D_{g} \bigl(x^{*},x^{k+1}\bigr), \quad \forall x^{*}\in S_{D}; $$
(ii) $\{x^{k}\}$ *is a bounded subset of* *C*;
(iii) $$\lim_{k\rightarrow\infty}D_{g}\bigl(x^{k+1},x^{k} \bigr)=0; $$
(iv) 12 $$ D_{g}\bigl(x^{k},x^{k+1}\bigr)\geq \frac{\alpha\alpha ^{2}_{k}}{L^{2}}(1-\sigma)^{2}D^{2}_{g}\bigl( \Pi^{g}_{C}\bigl(z^{k}\bigr),x^{k}\bigr). $$

### Proof

(i) Applying the definition of $D_{g}$, for all $x,y,z\in\mathbb{B}$, we have

13 $$ D_{g}(y,z)+D_{g}(z,x)-D_{g}(y,x)= \bigl\langle g'(z)-g'(x),z-y\bigr\rangle . $$

Taking $z=x^{k+1}$ and $x=x^{k}$ in (13), it follows from $x^{k+1}=\Pi ^{g}_{C\cap H_{k}}(x^{k})$ and Lemma 2.1 that

14 $$\begin{aligned}& D_{g}\bigl(y,x^{k+1}\bigr)+D_{g} \bigl(x^{k+1},x^{k}\bigr)-D_{g}\bigl(y,x^{k} \bigr) \\& \quad =\bigl\langle g'\bigl(x^{k+1}\bigr)-g' \bigl(x^{k}\bigr),x^{k+1}-y\bigr\rangle \leq0,\quad y\in C\cap H_{k}. \end{aligned}$$

Taking $y=x^{*}\in S_{D}$ in (14), we obtain

$$D_{g}\bigl(x^{k+1},x^{k}\bigr)\leq D_{g} \bigl(x^{*},x^{k}\bigr)-D_{g}\bigl(x^{*},x^{k+1} \bigr). $$

(ii) It follows from $D_{g}(x^{k+1},x^{k})\geq0$ and (11) that the sequence $\{D_{g}(x^{*},x^{k})\}$ is nonincreasing with lower bounds and hence is a converging sequence. This implies that $\{ D_{g}(x^{*},x^{k})\}$ is a bounded sequence. Using (H2), we obtain

$$D_{g}\bigl(x^{*},x^{k}\bigr)\geq\alpha \bigl\Vert x^{*}-x^{k} \bigr\Vert ^{2} \quad \text{for all } k. $$

Consequently, $\{x^{k}\}$ is a bounded sequence.

(iii) Using (11), we obtain

$$\sum^{\infty}_{i=0}D_{g} \bigl(x^{k+1},x^{k}\bigr)\leq\sum^{\infty }_{i=0} \bigl[D_{g}\bigl(x^{*},x^{k}\bigr)-D_{g} \bigl(x^{*},x^{k+1}\bigr)\bigr]\leq D_{g} \bigl(x^{*},x^{0}\bigr), $$

which implies that

$$\lim_{k\rightarrow\infty}D_{g}\bigl(x^{k+1},x^{k} \bigr)=0. $$

(iv) By (ii) the sequence $\{x^{k}\}$ is bounded and *T* is uniformly continuous on bounded subsets of $\mathbb{B}$, which by (H3), (H5), and Lemma 2.4 implies that $\{z^{k}\}$ is bounded, and thus, by Lemma 2.3, $\{\Pi^{g}_{C}(z^{k})\}$ is bounded. Consequently, $\{y^{k}\}$ is bounded. Taking into account the uniform continuity of *T*, we obtain that $\{T(y^{k})\}$ is also bounded, that is, there exists a positive number *L* such that

$$\bigl\Vert T\bigl(y^{k}\bigr) \bigr\Vert _{*}\leq L, \quad \forall k. $$

Then from (5) it follows that

$$\bigl\vert h_{k}\bigl(x^{k}\bigr)-h_{k} \bigl(x^{k+1}\bigr) \bigr\vert = \bigl\vert \bigl\langle T \bigl(y^{k}\bigr),x^{k}-x^{k+1}\bigr\rangle \bigr\vert \leq \bigl\Vert T\bigl(y^{k}\bigr) \bigr\Vert _{*} \bigl\Vert x^{k}-x^{k+1} \bigr\Vert \leq L \bigl\Vert x^{k}-x^{k+1} \bigr\Vert , $$

that is, $h_{k}$ is Lipschitz continuous on *C*. Combining Lemma 3.3 and Lemma 3.5, we obtain

$$ D_{g}\bigl(x^{k},x^{k+1}\bigr)\geq \frac{\alpha\alpha ^{2}_{k}}{L^{2}}(1-\sigma)^{2}D^{2}_{g}\bigl( \Pi^{g}_{C}\bigl(z^{k}\bigr),x^{k}\bigr). $$

 □

### Theorem 4.2

*Assume that* $S_{D}$ *is a nonempty set*, $T:\mathbb{B}\rightarrow \mathbb{B^{*}}$ *is uniformly continuous on bounded subset of* $\mathbb {B}$, *and* $g:\mathrm{B}\rightarrow R$ *satisfies* (H1)*–*(H5). *If* *C* *is a closed and convex subset of* *B* *and* $\{x^{k}\}$ *is an infinite sequence generated by Algorithm* 3.1, *then each weak accumulation point of* $\{ x^{k}\}$ *is a solution of* $\operatorname{VI}(T,C)$.

### Proof

Applying Theorem 4.1(iii) and (iv), we get

15 $$ \lim_{k\rightarrow\infty}\alpha_{k}D_{g} \bigl(\Pi ^{g}_{C}\bigl(z^{k}\bigr),x^{k} \bigr)=0. $$

Since $\mathbb{B}$ is a reflexive Banach space and $\{x^{k}\}$ is bounded by Theorem 4.1(ii), $\{x^{k}\}$ has at least one weak accumulation point. Let $x^{*}$ be any weak accumulation point of $\{ x^{k}\}$ such that the subsequence $\{x^{k_{i}}\}$ of $\{x^{k}\}$ weakly converges to $x^{*}$, that is, $x^{k_{i}}\rightharpoonup x^{*}$, $i\rightarrow\infty$, which implies by (15) that

16 $$ \lim_{i\rightarrow\infty}\alpha_{k_{i}}D_{g} \bigl(\Pi ^{g}_{C}\bigl(z^{k_{i}}\bigr),x^{k_{i}} \bigr)=0. $$

Then we prove that $x^{*}$ is a solution of $\operatorname{VI}(T,C)$ by discussing two cases.

*Case* 1: If $\limsup_{i\rightarrow\infty}\alpha _{k_{i}}> 0$, then exists a subsequence, without loss of generality, still recorded as $\{\alpha_{k_{i}}\}$, and a constant $\theta>0$ such that, for all *i*, we have $\alpha_{k_{i}}>\theta$. Therefore, using (16), we obtain

17 $$ \lim_{i\rightarrow\infty}D_{g}\bigl(\Pi ^{g}_{C}\bigl(z^{k_{i}}\bigr),x^{k_{i}} \bigr)=0. $$

It follows from Lemma 2.2 that

18 $$ \lim_{i\rightarrow\infty} \bigl\Vert \Pi ^{g}_{C} \bigl(z^{k_{i}}\bigr)-x^{k_{i}} \bigr\Vert =0. $$

Lemma 2.1 implies that

19 $$ \bigl\langle g'\bigl(z^{k_{i}} \bigr)-g'\bigl(\Pi ^{g}_{C}\bigl(z^{k_{i}} \bigr)\bigr),y-\Pi^{g}_{C}\bigl(z^{k_{i}}\bigr)\bigr\rangle \leq0,\quad \forall y\in C. $$

It follows from the definition of $z^{k_{i}}$ in Algorithm 3.1 that

20 $$ \bigl\langle g'\bigl(x^{k_{i}} \bigr)-g'\bigl(\Pi ^{g}_{C}\bigl(z^{k_{i}} \bigr)\bigr),y-\Pi^{g}_{C}\bigl(z^{k_{i}}\bigr)\bigr\rangle \leq\bigl\langle T\bigl(x^{k_{i}}\bigr),y-\Pi^{g}_{C} \bigl(z^{k_{i}}\bigr)\bigr\rangle ,\quad \forall y\in C. $$

This implies

21 $$ \bigl\langle g'\bigl(x^{k_{i}} \bigr)-g'\bigl(\Pi^{g}_{C}\bigl(z^{k_{i}} \bigr)\bigr),y-\Pi ^{g}_{C}\bigl(z^{k_{i}}\bigr)\bigr\rangle -\bigl\langle T\bigl(x^{k_{i}}\bigr),x^{k_{i}}-\Pi ^{g}_{C}\bigl(z^{k_{i}}\bigr)\bigr\rangle \leq\bigl\langle T\bigl(x^{k_{i}}\bigr),y-x^{k_{i}}\bigr\rangle . $$

Using (H3), (18), and the boundedness of $\{x^{k_{i}}\}$ and $\{\Pi ^{g}_{C}(z^{k_{i}})\}$, for all given $y\in C$, letting $i\rightarrow \infty$ in both sides of (21), we obtain that

22 $$ \liminf_{i\rightarrow\infty}\bigl\langle T\bigl(x^{k_{i}} \bigr),y-x^{k_{i}}\bigr\rangle \geq0. $$

Therefore, for any given $\varepsilon>0$, there exists a large enough positive integer *N*, such that, for $i\geq N$, we have

23 $$ \bigl\langle T\bigl(x^{k_{i}}\bigr),y-x^{k_{i}}\bigr\rangle +\varepsilon \geq0. $$

Note that $T(x^{k_{i}})\neq0$ by Lemma 3.4. Take $v^{k_{i}}\in\mathbb {B}$ such that $\langle T(x^{k_{i}}),v^{k_{i}}\rangle=1$. Then inequality (23) can be written as

24 $$ \bigl\langle T\bigl(x^{k_{i}}\bigr),y+\varepsilon v^{k_{i}}-x^{k_{i}}\bigr\rangle \geq0,\quad i\geq N, $$

which implies, using Lemma 2.5, that at least one of the following must hold:

25 $$ \bigl\langle T\bigl(y+\varepsilon v^{k_{i}}\bigr),y+ \varepsilon v^{k_{i}}-x^{k_{i}}\bigr\rangle \geq0,\quad i\geq N, $$

or

26 $$ \bigl\langle T\bigl(x^{k_{i}}\bigr),z-x^{k_{i}}\bigr\rangle \geq0, \quad \forall z\in C, i\geq N. $$

Inequality (26) implies that $x^{k_{i}}$ is a solution of $\operatorname{VI}(T,C)$, which contradicts $x^{k_{i}}\neq\Pi^{g}_{C}(z^{k_{i}})$. Thus inequality (25) must hold. Inequality (25) can be equivalently written

27 $$ \bigl\langle T(y),y-x^{k_{i}}\bigr\rangle \geq\bigl\langle T(y)- T\bigl(y+\varepsilon v^{k_{i}}\bigr),y+\varepsilon v^{k_{i}}-x^{k_{i}}\bigr\rangle -\varepsilon\bigl\langle T(y),v^{k_{i}}\bigr\rangle ,\quad i\geq N. $$

From the continuity of *T* and the boundedness of $\{x^{k_{i}}\}$, letting $\varepsilon\rightarrow0$, we obtain

28 $$ \bigl\langle T(y),y-x^{k_{i}}\bigr\rangle \geq0,\quad \forall y\in C. $$

Taking into account the fact that $x^{k_{i}}\rightharpoonup x^{*}$, $i\rightarrow\infty$, we obtain

29 $$ \bigl\langle T(y),y-x^{*}\bigr\rangle \geq0,\quad \forall y\in C, $$

that is, $x^{*}\in S_{D}$. It follows from $S_{D}\subset S$ that $x^{*}$ is a solution of $\operatorname{VI}(T,C)$.

*Case* 2: If $\lim_{i\rightarrow\infty}\alpha _{k_{i}}= 0$, then

30 $$ \lim_{i\rightarrow\infty}D_{g}\bigl(\Pi ^{g}_{C}\bigl(z^{k_{i}}\bigr),x^{k_{i}} \bigr)=0. $$

In fact, take

$$\bar{y}^{k_{i}}=\frac{\alpha_{k_{i}}}{\gamma}\Pi ^{g}_{C} \bigl(z^{k_{i}}\bigr)+\biggl(1-\frac{\alpha_{k_{i}}}{\gamma}\biggr)x^{k_{i}} $$

or, equivalently,

31 $$ \bar{y}^{k_{i}}-x^{k_{i}}=\frac{\alpha _{k_{i}}}{\gamma}\bigl( \Pi^{g}_{C}\bigl(z^{k_{i}}\bigr)-x^{k_{i}} \bigr). $$

From the boundedness of $\{\Pi^{g}_{C}(z^{k_{i}})-x^{k_{i}}\}$ and $\lim_{i\rightarrow\infty}\alpha_{k_{i}}= 0$ we obtain

32 $$ \lim_{i\rightarrow\infty} \bigl\Vert \bar {y}^{k_{i}}-x^{k_{i}} \bigr\Vert =0. $$

It follows from the definition of $\alpha_{k_{i}}$ that

33 $$ \bigl\langle T\bigl(x^{k_{i}}\bigr)-T\bigl( \bar{y}^{k_{i}}\bigr), x^{k_{i}}-\Pi^{g}_{C} \bigl(z^{k_{i}}\bigr)\bigr\rangle >\sigma D_{g}\bigl(\Pi ^{g}_{C}\bigl(z^{k_{i}}\bigr),x^{k_{i}}\bigr). $$

Using the uniform continuity of *T* on bounded sets of $\mathbb{B}$, $\sigma\in(0,1)$, and the boundedness of $\{\Pi^{g}_{C}(z^{k_{i}})\} $ and $\{x^{k_{i}}\}$, we obtain

34 $$ \lim_{i\rightarrow\infty}D_{g}\bigl(\Pi ^{g}_{C}\bigl(z^{k_{i}}\bigr),x^{k_{i}} \bigr)=0. $$

Next, applying a similar argument as in Case 1, we get the desired result. □

Now we can state and prove our main convergence result.

### Theorem 4.3

*Assume that* $S_{D}$ *is a nonempty set*, $T: \mathbb{B}\rightarrow \mathbb{B^{*}}$ *is uniformly continuous on bounded subset of* $\mathbb {B}$, *and* $g:\mathrm{B}\rightarrow R$ *satisfies* (H1)*–*(H5). *Let* *C* *is a closed and convex subset of* *B*. *Then any infinite sequence* $\{x^{k}\}$ *generated by Algorithm* 3.1 *strongly converges to a solution* *x̂* *of* $\operatorname{VI}(T,C)$.

### Proof

Let *x̂* be any weak accumulation point of $\{x^{k}\}$, and let $\{x^{k_{i}}\}$ be a subsequence of $\{x^{k}\}$ such that $x^{k_{i}}\rightharpoonup\hat{x}$, $i\rightarrow\infty$. By Theorem 4.2, *x̂* is a solution of $\operatorname{VI}(T,C)$. We next prove that the whole sequence $\{x^{k}\}$ strongly converges to *x̂*. Indeed, since *g* is strongly convex, we have

35 $$ \alpha \bigl\Vert x^{*}-x^{k_{i}} \bigr\Vert ^{2}\leq g\bigl(x^{*}\bigr)-g\bigl(x^{k_{i}}\bigr)- \bigl\langle g'\bigl(x^{k_{i}}\bigr),x^{*}-x^{k_{i}} \bigr\rangle . $$

The function *g* is lower semicontinuous and convex and, thus, weakly lower semicontinuous. Hence

36 $$ g\bigl(x^{*}\bigr)\leq\liminf_{i\rightarrow\infty}g \bigl(x^{k_{i}}\bigr). $$

Since $g'$ is uniformly continuous on bounded subsets of $\mathbb{B}$ and $\{x^{k_{i}}\}$ is bounded, by Lemma 2.4 we get that $\{ g'(x^{k_{i}})\}$ is bounded. From (35) and (36) we have

$$\begin{aligned} \limsup_{i\rightarrow\infty}\alpha \bigl\Vert x^{*}-x^{k_{i}} \bigr\Vert ^{2} \leq& \limsup_{i\rightarrow\infty}\bigl(g \bigl(x^{*}\bigr)-g\bigl(x^{k_{i}}\bigr)-\bigl\langle g'\bigl(x^{k_{i}}\bigr),x^{*}-x^{k_{i}}\bigr\rangle \bigr) \\ =& g\bigl(x^{*}\bigr)-\liminf_{i\rightarrow\infty}g \bigl(x^{k_{i}}\bigr)-\liminf_{i\rightarrow\infty}\bigl\langle g'\bigl(x^{k_{i}}\bigr),x^{*}-x^{k_{i}}\bigr\rangle . \\ \leq& g\bigl(x^{*}\bigr)-g\bigl(x^{*}\bigr)-\liminf _{i\rightarrow\infty}\bigl\langle g'\bigl(x^{k_{i}} \bigr),x^{*}-x^{k_{i}}\bigr\rangle =0, \end{aligned}$$

which implies that

37 $$ \lim_{i\rightarrow\infty} \bigl\Vert x^{*}-x^{k_{i}} \bigr\Vert =0. $$

Since *g* and $g'$ are uniformly continuous on bounded subsets of $\mathbb{B}$, $\{x^{k_{i}}\}$ is bounded, and

$$ D_{g}\bigl(x^{*},x^{k_{i}}\bigr)=g \bigl(x^{*}\bigr)-g\bigl(x^{k_{i}}\bigr)-\bigl\langle g'\bigl(x^{k_{i}}\bigr),x^{*}-x^{k_{i}}\bigr\rangle . $$

Letting $i\rightarrow\infty$ in this inequality and combining this with (37), we obtain

38 $$ \lim_{i\rightarrow\infty}D_{g}\bigl(x^{*},x^{k_{i}} \bigr)=0. $$

Applying the convergence of the whole sequence $\{D_{g}(x^{*},x^{k})\} $, we get

39 $$ \lim_{k\rightarrow\infty}D_{g}\bigl(x^{*},x^{k} \bigr)=0. $$

From Lemma 2.2 it follows that

$$ \lim_{ k\rightarrow\infty} \bigl\Vert x^{*}-x^{k} \bigr\Vert =0, $$

that is, the whole sequence $\{x^{k}\}$ strongly converges to $x^{*}$. □

## Conclusion

In this paper, by the Bregman projection we extend a double projection algorithm proposed by Solodov and Svaiter [7] for solving variational inequalities from Euclidean spaces to Banach spaces. Under the assumptions of $S_{D}\neq\emptyset$, uniform continuity and quasimonotonicity, we prove that the whole sequence generated by the proposed method is strongly convergent to the solution of the variational inequalities, and our proof techniques are different from those presented in [14–17].
